# Sliding and Lower Limb Mechanics during Sit-Stand-Sit Transitions with a Standing Wheelchair

**DOI:** 10.1155/2014/236486

**Published:** 2014-07-06

**Authors:** Yu-Sheng Yang, Ming-De Chen, Wei-Chien Fang, Jyh-Jong Chang, Chang-Chih Kuo

**Affiliations:** ^1^Department of Occupational Therapy, College of Health Science, Kaohsiung Medical University, 100 Shih-Chuan 1st Road, Kaohsiung 80708, Taiwan; ^2^Department of Rehabilitation Medicine, Kaohsiung Municipal United Hospital, Kaohsiung City, Taiwan

## Abstract

*Purpose.* This study aimed to investigate the shear displacement between the body and backrest/seat, range of motion (ROM), and force acting on the lower limb joints during sit-stand-sit transitions by operating an electric-powered standing wheelchair. *Methods and Materials.* The amounts of sliding along the backrest and the seat plane, ROM of lower limb joints, and force acting on the knee/foot were measured in twenty-four people with paraplegia. *Results.* Without an antishear mechanism, the shear displacement was approximately 9 cm between the user's body and the backrest/seat surfaces. During standing up, the user's back slid down and the thigh was displaced rearward, but they moved in opposite directions when wheelchair sat back down. A minimum of 60 degrees of ROM at the hip and knee was needed during sit-stand-sit transitions. The maximal resultant forces acting on the knee restraints could reach 23.5% of body weight. *Conclusion.* Sliding between the body and backrest/seat occurred while transitioning from sitting to standing and vice versa. A certain amount of ROM at lower limb joints and force acting on the knee was necessitated during sit-stand-sit transitions. Careful consideration needs to be given to who the user of the electric powered standing wheelchair is.

## 1. Introduction

Standing is a routine activity of daily living (ADL) that allows individuals not only to talk and socialize with others, but also to perform many regular and important ADLs, such as cooking, grooming, or reaching objects. For people with paraplegia, standing with the help of a standing frame or a standing wheelchair helps to increase functional independence and improves an individual's physical and psychological well-being [1]. A standing wheelchair is an assistive device that enables people with paraplegia to achieve an upright posture and perform standing activities. A standing feature integrated into a wheelchair base allows the user to obtain a standing position without the need to transfer from the wheelchair. A mechanical or electromechanical system manipulated via the wheelchair's controller moves the seat surface from horizontal into a vertical or obliquely sloping position to anterior inferiorly position while maintaining vertical position of the legrest and backrest, thus extending the hip and knee joints. A full vertical standing position can be achieved directly from sitting, or through gradual angle changes from a supine position, or a combination of these positions [2]. People with paraplegia may use a standing wheelchair to stand up for a variety of health-related reasons, such as increasing their range of motion [3, 4], maintaining bone density [5, 6], improving circulation [7], decreasing spasticity [8, 9], preventing pressure sores [7, 9], and improving bowel and bladder function [4, 7, 9].

Moreover, rising from sitting to standing and its reverse are necessary and functionally important activities. While using a standing wheelchair, each sit-stand-sit movement cycle consisted of two phases: a rising phase during which the subject rose passively by a standing mechanism from the seated position to the standing position, and a descending phase during which the subject descended from standing to the seated position. These two phases in the sit-stand-sit movement cycle are discrete. During the transition from sitting to standing, the seat and knee block of the wheelchair which supports the user are often rotated about the pivot points parallel to the user's hip and knee joint, causing the user to assume a standing position [10]. If the joints of the wheelchair do not follow the anatomical paths of the user, the sliding will occur. On the contrary, sitting down transition is performed with the assistance of gravity (downward movement). The forward trunk rotation into flexion required to sit down was operated by lowering the seat while keeping the backrest in a rearwardly inclined vertical position. As a result, large sliding displacement could occur and these shear forces will be developed because these forces between the user and the backrest/seat usually prevent the user from sliding back up to the original position. Large shear forces in the backrest and seat could lead to the development of pressure ulcer [11]. Study has shown that, without an antishear mechanism, up to 11 cm of displacement can take place between the person's back and the wheelchair's backrest while using powered reclining wheelchair [12]. But there were a few studies addressing these displacements with respect to the backrest and seat plane on the standing wheelchair. Therefore, it is worth investigating possible shear displacements with the use of a standing wheelchair.

In order to secure the standing mechanism, the larger force would be applied to the lower extremities, especially at knee joint, while using knee block placed just anteriorly to the knees. Excessive force exerted against the knee joint might be at high risk for a bone fracture [13]. So far, to the best of the author's knowledge, no studies investigated the magnitude of force over the knee block during transition of standing with a standing wheelchair. Furthermore, when individuals with paraplegia are unable to stand independently due to increased muscle tone, weakness, or imbalance, they are at risk of shortening (contracture) of the muscles which bend the hip (e.g., iliopsoas), those which straighten the hip and bend the knee (e.g., hamstrings), and the calf muscle which bends the knee and ankle. While being not a substitute for therapy, the user of standing wheelchair may benefit from partial weight bearing even if he or she already has fixed contractures of the lower extremities. Many people in wheelchairs have limited access to therapy or caregivers who can provide the necessary amount of ranging. Standers integrated with the wheelchair base allow them to perform this important activity on their own and with higher frequency [2]. However, in order to avoid overstretching contracted muscles, it is critical to know the range of motion (ROM) of lower extremity joints during sit-to-stand with a standing wheelchair. Most clinicians are familiar with requirements for obtaining proper static seating posture in a manual wheelchair; few are familiar with dynamic seating posture in a standing wheelchair. As standing wheelchairs are more complex than most manual or electrically power wheelchair, knowing the prerequisite ROM of these joints could help the clinician to make decisions prior to selecting and fitting the user on it.

For these reasons, the purpose of this study was to measure the shear displacement between the body in people with spinal cord injuries and backrest/seat during both the transition of standing up and sitting down by operating an electric powered wheelchair. We also were interested in investigating the ROM of low limb joint and force acting on the knee restraint to determine the prerequisite joint range and the magnate of forces against the knee during sit-to-stand-to-sit cycles. Due to different mechanical and postural conditions, we further hypothesized that standing up and sitting down would affect shear displacement and force acting on the knee block in different ways.

## 2. Materials and Methods

### 2.1. Participants

Twenty-four people (17 men and 7 women) with thoracic or lumbar spinal cord injuries (American Spinal Injury Association Impairment Scale type A or B) participated in this study. None of them had ever utilized an electric standing wheelchair before. The mean age was 41.60 ± 11.33 years old, mean weight was 63.92 ± 12.85 kg, and mean length of leg was 81.39 ± 6.84 cm, respectively. They have neither fixed contracture nor skeletal deformities of lower extremities. Known cases of osteoporosis were also excluded. All participants provided written informed consent in accordance with the procedures approved by the Institutional Review Board of the Kaohsiung Medical University Hospital prior to participation in the study.

### 2.2. Instrumentation

**Figure 1:**
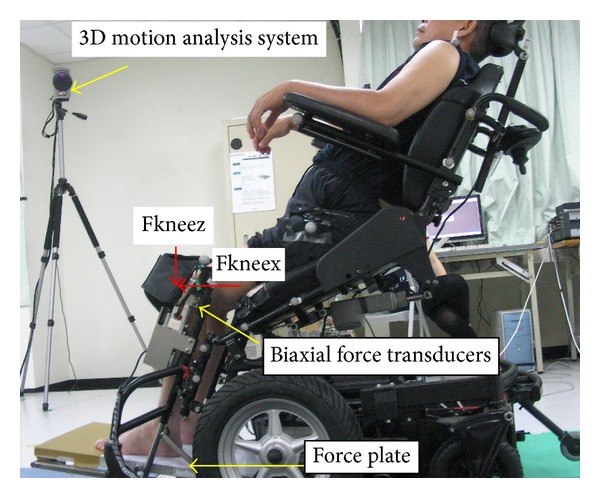
Experimental setting.

An electrically powered standing wheelchair (Model LY-ESB240, Comfort Orthopedic Co. Ltd., Chia-Yi, Taiwan) was used in this study. The overall dimension of length with footrests, seat height, and width were 113.5 cm, 56 cm, and 45.7 cm, respectively. The maximal angle to hoist a user upright was 80 degrees. No particular shear reduction technology either by back or seat frames was employed in this model. Each participant was required to wear tight fitting clothing (e.g., spandex shorts and sleeveless shirt) to prevent problems with “marker plucking.” Reflective markers were placed bilaterally to the surface of the skin over the following bony landmarks: acromion process, great trochanter, lateral epicondyle of the femur, the medial and lateral malleoli, and the fifth metatarsal base. Moreover, eight additional markers were placed on both sides of the backrest frame and seat frame of the wheelchair to determine the backrest and seat reference plane. Three-dimensional marker trajectory data were measured using a six-camera motion analysis system (Qualisys Medical AB, Göteborg, Sweden) at a sampling rate of 120 Hz (Figure 1).

The biaxial force transducers (Model BFT100, Junzhi Engineering Co., Ltd., Kaohsiung, Taiwan) were mounted bilaterally at the knee block to measure the anteroposterior and vertical force acting at the knee restraint with a 10 cm wide nylon strap (Figure 1). Normal ground reaction forces at the footrest were measured using a force plate with a sample frequency of 120 Hz (Model 9286AA, Kistler Instrument, Amherst, NYA).

### 2.3. Experimental Protocol

At the beginning of testing, the length of the legrest was adjusted when the subject sat comfortably in the wheelchair so that the thighs rested parallel to the seat surface with the foot comfortably placed on the footrest. Afterwards, each subject was asked to transition from sit-to-stand and then back to sit through the operation of the electric powered standing wheelchair. The experimental trail would be stopped immediately for safety concerns and redone again if the participant could not continue sit-to-stand and stand-to-sit transitions, due to orthostatic hypotension or danger of sliding out of the wheelchair. Data were recorded throughout three complete sit-to-stand-to-sit cycles. Between each cycle, a one-minute break was provided.

### 2.4. Data Analysis

Displacements between the body and wheelchair backrest/seating surface represent the degrees of sliding along the backrest (BS) and sliding along the seat (SS). Therefore, BS was calculated as the distance between the acromion and the backrest frame marker on the backrest reference plane, and SS was defined as the distance between the greater trochanter and seat frame marker on the seat reference plane [14]. Positive values of BS indicated that the location of acromion was displaced upward along the backrest, whereas negative values indicated downward displacements from the initial position. Positive values of SS indicated rearward displacements of the greater trochanter along the seat plane, whereas negative values indicate forward displacements from the initial position. The sliding along the backrest and along the seat at left and right side was then averaged to represent the shear sliding for these two surfaces, respectively. When going through sit-to-stand or stand-to-sit transitions, the body and thigh may shift up/down or fore/back. Therefore, the cumulative values of BS and SS were used to represent the sum of shear displacements during sit-to-stand and stand-to-sit transitions. The difference between the maximum and minimum displacement of BS and SS was calculated as the range of BS and SS to describe the possible range of shear displacements. In addition, from the position of the markers three joint angles were calculated and analyzed: (1) the hip angle between the trunk (link between acromion process and great trochanter) and the thigh (link between the great trochanter and the lateral femoral epicondyle), (2) the knee angle between the thigh and the shank (link between the lateral femoral epicondyle and the external malleolus), and (3) the ankle angle between the shank and foot (link between the external malleolus and fifth metatarsal base). Joint ROM for the hip, knee, and ankle were calculated as the difference between the maximum and minimum joint angle. The magnitudes of force acting on the knee restraint, normal ground reaction force at the ankle joint, were also recorded and normalized with respect to individual body weight. Afterwards, the mean value for above kinematic and kinetic variable across three trials was computed using MATLAB (The MathWorks, Natick, MA) software.

### 2.5. Statistical Analyses

Descriptive statistics were used to describe selected dependent variables. The comparison of the BS, SS, ROM of low extremity joints, and forces acting on the knee restraint between sit-to-stand and stand-to-sit transitions was made using a paired* t*-test. All statistical tests were performed using the SPSS for Windows 13.0 (SPSS Inc., Chicago, IL) software package. The level of significance was set to 0.05.

## 3. Results

### 3.1. Relative Shear Displacement during Sit-to-Stand-to-Sit Transition

**Figure 2:**
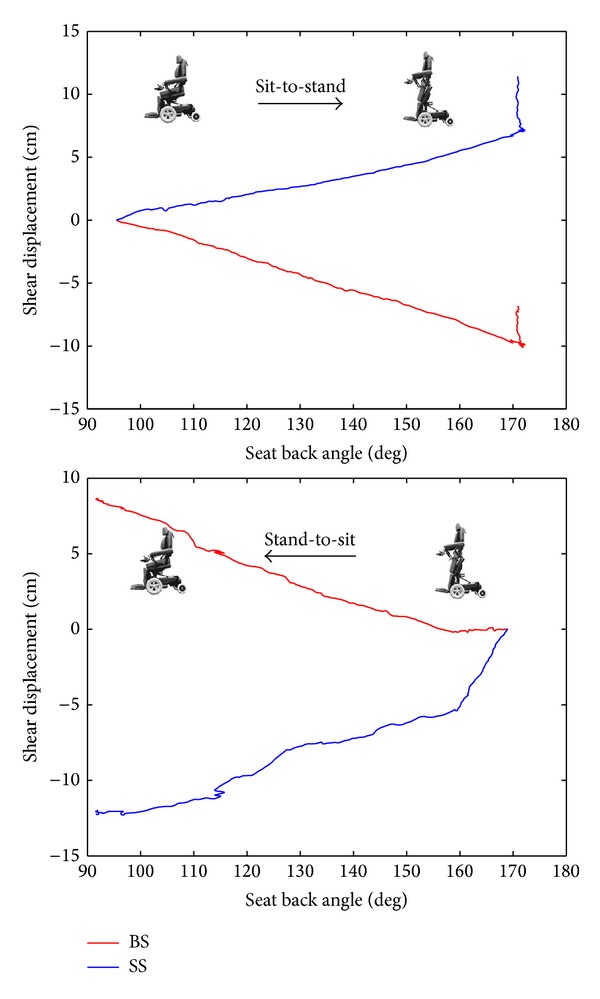
Representative plot (subject number 14, Trial 1) of shear displacement of the user's body sliding along the backrest (BS) and sliding along the seat (SS) as the seat-back angle of the standing wheelchair transformed from sit-to-stand and vice versa.

A representative plot of the shear displacements for the back to backrest and thigh to seat was shown in Figure 2. During sit-to-stand transition, the average BS values of each subject were negative, indicating that the upper body was displaced downward, but positive values of SS indicated rearward displacement of the greater trochanter along the seat plane. On the contrary, positive values of BS with negative values of SS were found during a stand-to-sit transition. It was shown that stand-to-sit displaced the upper body into upward direction and thighs into forward direction (Figure 2). The statistical analysis comparing sit-to-stand and stand-to-sit transitions revealed no significant differences in the cumulative values of BS (*P* = 0.53) and SS (*P* = 0.07), but significant differences in the range of BS and SS were found (*P* < 0.05). The range of shear displacements along the backrest plane (BS) was significantly larger during sit-to-stand transition (*P* < 0.01). On the other hand, during stand-to-sit transition the range of shear displacements along the seat plane (SS) was significantly larger (*P* = 0.01).

### 3.2. The Features of Joint Kinematics during Sit-to-Stand-to-Sit Transition

**Figure 3:**
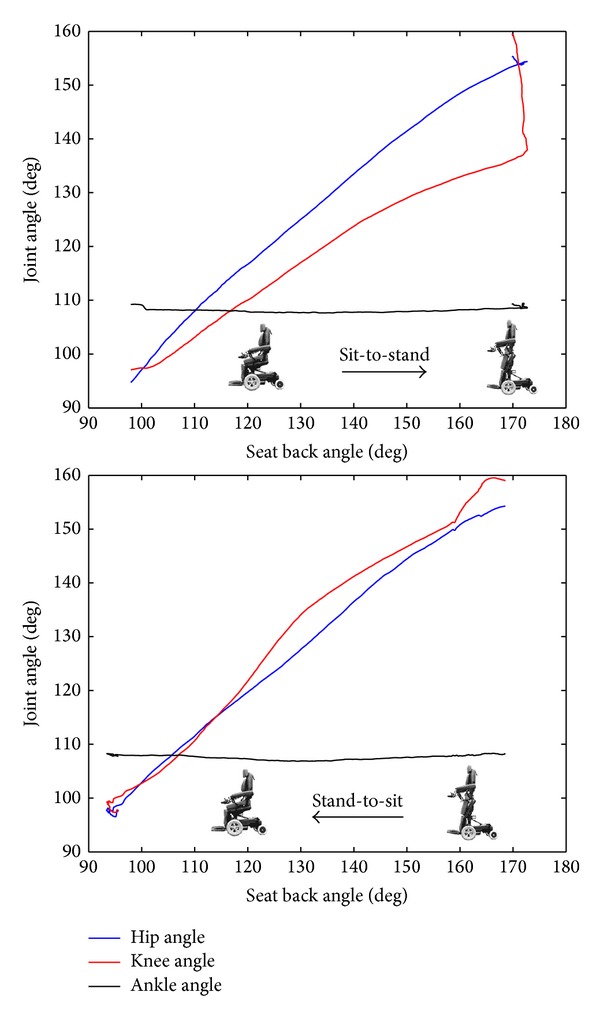
Representative plot (subject number 10) of lower extremity joint angle motion as the seat-back angle of the standing wheelchair transformed from sit-to-stand and vice versa.

There are no significant differences in the ROM of hip, knee, and ankle joint when comparing sit-to-stand and stand-to-sit transitions (Table 1). The average ROM of hip and knee joint was 62.1 and 60.8 degrees, respectively, but much less ROM of ankle joint (2~3 degrees) was found. During sit-to-stand transition, the hip and knee joint angle increased in accordance with an increase in the seat-to-back angle. Moreover, the knee angle still increased despite the fact that the backrest was reclined to a flat position (Figure 3). On the contrary, the hip and knee joint angle both decreased with decreasing seat-to-back angle during a stand-to-sit transition (Figure 2). No significant changes in ankle ROM were detected during sit-to-stand-to-sit transition.

### 3.3. The Force Acting on the Knee Restraint during Sit-to-Stand-to-Sit Transition

**Figure 4:**
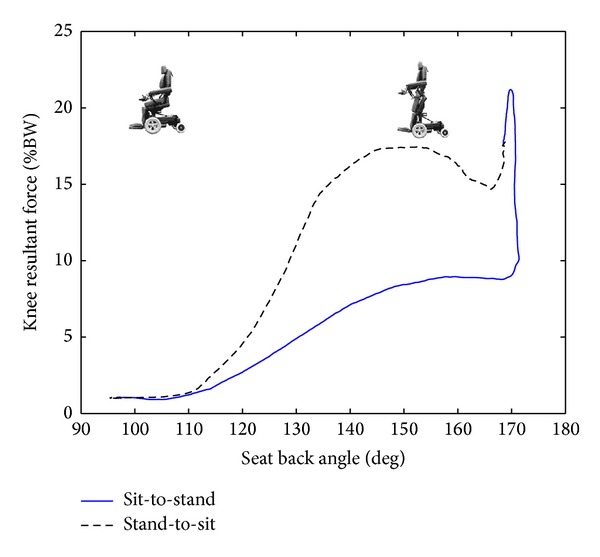
Representative plot (subject number 10) of the resultant forces acting on the knee as the seat-back angle of the standing wheelchair transformed from sit-to-stand and vice versa.

Results showed that the resultant forces acting on the knee restraints were significantly larger during sit-to-stand transition than during stand-to-sit transition (*P* = 0.01). During sit-to-stand transition, the resultant forces increased as the knee was extended (Figure 4). At the standing position, large force was applied on the knee to keep legs straight while standing, but by lowering the seat with increasing the knee flexion angle, the resultant forces acting on the knee were decreased when sitting back. Then, a more detailed analysis comparing separately anterior-posterior and upward-downward forces between these two transitions revealed that the maximal and average anterior force, which are against the knee restraint, were significantly greater during sit-to-stand transition (*P* < 0.01). But when moving back to sit the average downward forces were significantly larger (*P* = 0.01). No significant difference in the maximal downward forces was found between these two transitions (*P* = 0.19). Furthermore, while standing up in a standing wheelchair, an average 74.57 ± 13.67% of body weight by the user was attained onto the feet.

## 4. Discussion

Standing wheelchairs are confronted with the problem that relative displacements between the backrest/seat surfaces and the wheelchair user's body can occur during standing up or sitting down. A relative displacement between the wheelchair and the user's body is produced when the axis of rotation of the hinge mechanism does not correspond to the axis of rotation of the hip/knee joint. In reality, the situation is somewhat more complex because these anatomical joints somehow do not perform a pure rotational movement. Our results showed that the range of shear displacements were up to 8.7 cm for between the user's body and the wheelchair backrest/seat surfaces during sit-to-stand. When the standing wheelchair transformed from sitting to standing, the user's back slid down, and the greater trochanter was displaced rearward relative to the seat surface. On the other hand, when wheelchair sat back down, up to 7 cm and 10 cm of displacement could take place between the user's body and the wheelchair's backrest and seat surface, respectively.

Specifically, during transition from sitting to standing with opening the seat-to-back angle, standing up was performed with the seat lift mechanism being against gravity. The gravity would cause the user's body to slide down and forward along backrest and seat plane. If the sliding happened without any other restrictions, the user was likely to slide and fall out of the wheelchair. Therefore, the knee restraint which was placed anteriorly to the knees limited forward movement of the thighs as a “block” to prevent sliding and helped to reposition the knee from a flexed position to a straight position while standing. When standing with legs straight, this will further prevent the user sliding down along backrest and seat plane. As a result, the acromion slid down from the initial position in the beginning and then rebounded upward due to keeping the knee straight. At the same time, the greater trochanter was also slid rearward, close to the backrest when the wheelchair raised users from sitting to standing as shown in Figure 2.

On the contrary, during the transition from standing to sitting with closing the seat-to-back angle, the backrest was more likely going to a squeeze posture and moved the user into upward and forward direction. The upward displacement of acromion and forward displacement of greater trochanter, in consequence, could be found, as shown in Figure 2. Furthermore, there was more significant forward displacements of the greater trochanter than acromion in upward direction. When the standing wheelchair tried to move back into the sitting position, the act of moving the backrest upright often tried to push the user out of the seat even further unless the user was repositioned. No matter the transition from sitting to standing positions or vice versa, relative displacements between the body's position with respect to the backrest and seat surface was attributed to this dynamic posture change. Because the backrest and seat of the wheelchair slid against the user's back and thigh when standing and returning to an seated position, the use of any type of shear reduction technology on these two surface planes might reduce the shear displacements and further prevent the risk of skin breakdown and pressure sores.

The standing wheelchair usually provided three-point stabilization in a fully upright or vertical position, with supports at the lower torso, hips, and knees. A backrest upheld the lower torso, a seat lift supported hip posteriorly, and knee pads stabilized the knees anteriorly [15]. This standing mechanism allowed individuals with complete paralysis to stand passively and subsequently load their lower extremity. However, there were a few limitations as to who can use a standing wheelchair. The biggest was that the user's legs and joints must be able to support the user's torso weight without pain, discomfort, or doing any damage and that the user's hips and knees must have a certain range of motion as they would be extended/flexed during use. Our results showed that a minimum of 60 degrees of ROM in the hip and knee with few degrees of ankle ROM was required for safety of operation of a standing wheelchair. Since standing wheelchairs are powerful devices, they extended a user's hip and knee joint mechanically to provide posture change, but the amount of extension has to be limited, especially in the case of a client without sensation who would be unable to detect and indicate harmful stretches. It may cause harm if attempting to overstretch contracted muscles within limited ROM of hip and knee joint. Based on our findings, it was recommended that at least 60 degrees of ROM in the hip and knee was necessitated in a standing wheelchair. Less ROM of the ankle joint was wanted.

Our results also showed that the maximal resultant forces acting on the knee restraints could reach 23.5% of BW and were significantly larger during sit-to-stand transition than stand-to-sit transition. Furthermore, the magnitudes of force in anterior direction were also significantly larger. This indicated that knee restraints were used as a block to secure the knee and applied pressure to the upper shin as the wheelchair mechanisms worked in concert to raise the body to the standing position. The user was raised by a movement which was produced by the reaction force at the knee and the raising seat. On the contrary, during transitioning from standing to sitting, the knee restraints were more likely to support lower limbs and prevent accidental sliding downward. Therefore, more downward forces were observed, as compared with sit-to-stand transition. Regardless of whether sitting or standing in a standing wheelchair, knee blocking is critical and necessary to keep the knees straight without buckling. It was not possible to stand up or move back to sit without the knee restraints.

Many wheelchair users experience significant reduction in bone mineral density due to the lack of weight bearing which results in osteoporosis and a risk of fractures. Therefore, it is necessary to align the pivot mechanism for the knees with the center of joint rotation for the user's knee. Misalignment can place stress on the knee and tibia, which may lead to a fracture or joint laxity [13]. Our results showed that the average maximal resultant forces acting on the knee could reach 24% of BW (the range of 23.5% BW~19.6% BW). Dudley-Javoroski and Shields reported that in the first year post-SCI, between 15 and 35 percent of bone mineral density was lost in the region of the knee joint at greatest risk for fracture: the distal femur and proximal tibia [16]. Hence, it would more likely cause fracture around the knee joint if the standing wheelchair user had a lower bone mineral density. Careful consideration needs to be given to who the user of the standing wheelchair is.

Standing wheelchairs also promote weight bearing, which is essential to maintain bone density in the lower extremities. Our results indicated that an average weight-bearing load of 75% of body weight could be reached while standing upright in this model of standing wheelchair. This finding was consistent with previous studies using other standing devices. Although the amount of weight distributed through the lower limbs could vary depending on the standing device used, previous studies showed that weight bearing ranged from 68% to 85% of body weight [17, 18]. Ideally, it was expected that most weight bearing by the user of the standing wheelchair should be reached in the right circumstances. However, as sliding occurred between the user's body and backrest/seat plane, it was more challenging to achieve full hip and knee extension in a standing wheelchair than in a standing frame. In such situations, more weight was translated anteriorly into the proximal tibia against the knee restrains and posteriorly at the pelvis against the seat surface. Consequently, partial weight bearing was borne by the combination of the seating surface and knee restrains as found in our data. Moreover, during sit-to-stand transition, the participants in our study were asked to relax and rest their arms on the armrest for comfort. It was possible that the participants may grasp the armrest to particularly support their weights for stability during standing. A similar phenomenon was observed by Bernhardt et al. They indicated that supporting the arms on the standing frame tray while standing reduced the weight bearing by approximately 10% body weight. So, higher percentage of weight bearing could be obtained while standing in the standing wheelchair with their arms unsupported. Nevertheless, a large proportion of their body weight (approximately 75%) was still borne through the lower limbs even though their arms were resting on the armrest.

The scope of this study was limited because only one particular model of powered standing wheelchair was tested. There are numerous types of standing wheelchairs available on the market, including a manual standing wheelchair, a manual wheelchair with powered standing assist, or a fully powered wheelchair. Many manufactures now incorporate reclining mechanisms into the standing wheelchair mechanisms that are designed to eliminate sliding displacement during sit-stand-sit transition. Different models of wheelchair may incorporate these features including tilt-in-space, reclining, standing, and even elevating seat. The combination of these features might provide a smooth and low shear transition between standing and seating functions. Our study only demonstrated the possible shear displacements without implementing shear reduction technologies. Further research is needed to measure the effect of these shear reduction technologies for reducing shear and friction forces between the user's body and backrest/seat surface.

## 5. Conclusions

In spite of the numerous benefits, a standing wheelchair might be contraindicated without appropriate assessment. Our results showed that the shear displacement occurred between the user's body and the backrest/seat surfaces when using a stand-up function of the powered standing wheelchair. The shear forces from the backrest and seat surface while transitioning from sitting to standing and vice versa could result in a pressure sore. Furthermore, a user with fixed contractures of the lower extremities or existing osteoporosis was not an appropriate candidate for a standing wheelchair. Clinical practitioners should be aware of these contraindications and precautions.

## Figures and Tables

**Table 1:** Measurement of sliding along the backrest and seat plane, ROM of low extremities, and forces acting on the knee restraint between sit-to-stand and stand-to-sit transitions.

	Sit-to-stand	Stand-to-sit	*P* value
Cumulative BS (cm)	−4.84 ± 3.22	4.68 ± 3.57	0.53
Range of BS (cm)	8.72 ± 4.87	6.82 ± 4.04	<0.01∗
Cumulative SS (cm)	8.66 ± 2.87	−9.33 ± 3.25	0.07
Range of SS (cm)	8.83 ± 3.21	10.05 ± 3.31	0.01∗
ROM of hip joint (degrees)	61.78 ± 11.59	62.57 ± 9.74	0.59
ROM of knee joint (degrees)	60.66 ± 9.02	61.09 ± 7.82	0.71
ROM of ankle joint (degrees)	3.24 ± 1.91	3.14 ± 1.03	0.78
Maximal of resultant force (%BW)	23.51 ± 8.93	19.61 ± 8.04	<0.01∗
Average of resultant force (%BW)	9.58 ± 3.04	8.44 ± 3.27	0.01∗
Maximal anterior force (%BW)	22.98 ± 8.37	18.62 ± 8.09	<0.01∗
Average anterior force (%BW)	9.07 ± 3.02	7.29 ± 3.32	<0.01∗
Maximal downward force (%BW)	6.65 ± 2.67	7.48 ± 3.45	0.19
Average downward force (%BW)	2.04 ± 1.22	3.00 ± 2.00	0.01∗

*P < 0.05.
